# Exploration of Polysaccharides from *Phyllanthus emblica*: Isolation, Identification, and Evaluation of Antioxidant and Anti-Glycolipid Metabolism Disorder Activities

**DOI:** 10.3390/molecules29081751

**Published:** 2024-04-12

**Authors:** Peng Guo, Meng Chen, Wenzhao Wang, Qiuyun Li, Xinyu Chen, Jiayue Liang, Yiyang He, Yanli Wu

**Affiliations:** Department of Organic Chemistry, College of Pharmacy, Harbin Medical University, Harbin 150081, China

**Keywords:** polysaccharide, purification, identification, anti-glycolipid metabolism disorder activity

## Abstract

*Phyllanthus emblica* is a natural medicinal herb with diverse bioactivities. Certain extracts from this herb have been confirmed to possess anti-glycolipid metabolic disorder activity. To further develop its utility value and explore its potential in combating glycolipid metabolic disorders, we designed a series of experiments to investigate the structure, antioxidant activity, and anti-glycolipid metabolic disorder activity of *Phyllanthus emblica* polysaccharides. In this study, we extracted and purified polysaccharides from *Phyllanthus emblica* and thoroughly analyzed their structure using various techniques, including NMR, methylation analysis, and surface-enhanced Raman spectroscopy. We investigated the hypolipidemic and anti-glycolipid metabolism disorder activity of *Phyllanthus emblica* polysaccharides for the first time utilizing oleic acid (OA) and advanced glycation end products (AGEs) as inducers. Additionally, the antioxidant activity of *Phyllanthus emblica* polysaccharides was assessed in vitro. These findings lay the groundwork for future investigations into the potential application of *Phyllanthus emblica* polysaccharides as an intervention for preventing and treating diabetes.

## 1. Introduction

According to epidemiological studies, cardiovascular disease (CVD) will continue to be a medical security burden in China and the world for a long time [1,2]. Although the prevention and treatment of CVD have improved to some extent due to advancements in medical conditions and living standards, its incidence rate and mortality remain high [3]. Previous studies have shown that hyperlipidemia and diabetes play crucial roles in the generation and development of CVD [4]. Hyperlipidemia is primarily characterized by elevated levels of total cholesterol (TC), triglycerides (TG), and low-density lipoprotein cholesterol (LDL-C), along with reduced levels of high-density lipoprotein cholesterol (HDL-C). Meanwhile, diabetes is primarily associated with insulin resistance [5]. Generally, patients with CVD may concurrently experience varying degrees of dyslipidemia and abnormal glucose metabolism, a condition referred to as glycolipid metabolism syndrome [6].

Advanced glycosylation end products are usually produced by non-enzymatic glycosylation caused by hyperglycemia; they are a set of isomeric metabolites characterized by significant toxicity [7,8,9]. AGEs are difficult to remove in the body, are closely related to the occurrence and development of CVD, and are considered to be an important cause of microvascular complications and lipid mass spectrometry changes in diabetes [10].

At present, symptomatic treatment is often used in clinics to treat disorders of glucose and lipid metabolism. On the one hand, statins are used to reduce cholesterol, and fibrates are used to reduce triglycerides and increase high-density lipoprotein [11,12], such as Simvastatin, bezafibrate, etc. On the other hand, enhancing the body’s sensitivity to insulin and coordinating with α-glucosidase inhibitors can help treat glucose metabolism disorders [13,14]. However, the above drugs all have obvious side effects. Hypolipidemic drugs made from statins and fibrates may lead to the dissolution of striated muscle tissue [15], while thiazolidinedione has strong hepatotoxicity [16]. Natural drugs have the advantages of low toxicity and good long-term efficacy, and they have become a popular direction for the development of drugs to treat anti-glycolipid metabolism disorder [17].

*Phyllanthus emblica* has a long history of use as a traditional Chinese medicine and as food [18,19]. It was first seen in “Southern Vegetation” and used by the traditional medical systems of 17 countries, including India, Iran, and Afghanistan [20,21]. It was proven that it has beneficial effects, such as anti-tumor [22,23,24], anti-inflammatory [25,26], antioxidation [27], etc. The present experiment focuses on the phenolic acids and flavonoids of *Phyllanthus emblica*; there is little research on its polysaccharides. In this experiment, the water-soluble neutral *Phyllanthus emblica* polysaccharide was extracted and separated, and its structure was identified. We determined the antioxidant activity of *Phyllanthus emblica* polysaccharides through in vitro experiments and comprehensively evaluated their anti-glycolipid metabolism disorder activity.

## 2. Results and Discussion

### 2.1. Preparation and Physicochemical Property Analysis of PEP-1-1

Figure S1A illustrates the extraction and purification processes of PEP. Water extraction and alcohol precipitation were used to extract crude polysaccharide (cPEP) from *Phyllanthus emblica*. After eluted it with water on the ion exchange column, the neutral fraction that demonstrated the highest yield (PEP-1) was chosen to be purified via DEAE-52 column chromatography. The yield of purified products is shown in Table S1. The elution curve of PEP-1 is displayed in Figure S1B. The polysaccharide PEP-1-1 was isolated and exhibited a single symmetric peak (Figure S1C).

The average Mw of PEP-1-1 was calculated to be 29.3 kDa based on the standard curve (lgMw-RT: y = −0.287x + 7.4773, R^2^ = 0.9905). Furthermore, PEP-1-1 contained 91.23% total sugar, 0.35% protein, and 6.22% uronic acid, suggesting that PEP-1-1 was a neutral polysaccharide. The ultraviolet absorption spectrum of PEP-1-1 showed that no discernible absorption peaks were observed at 260 nm and 280 nm in Figure S1D, indicating exceedingly low levels of protein and nucleic acid.

In order to further characterize the structure of the polysaccharides, we detected the particle size and ζ- potential of PEP-1-1 using a Marvin particle size analyzer. The results showed that the particle size of PEP-1-1 in the aqueous solution was 1093 nm, with a ζ-potential of −4.22 mV, further confirming that PEP-1-1 is a neutral polysaccharide. The chemical composition and molecular properties of PEP-1-1 was showed in Table 1.

### 2.2. Monosaccharide Composition

**Figure 1:**
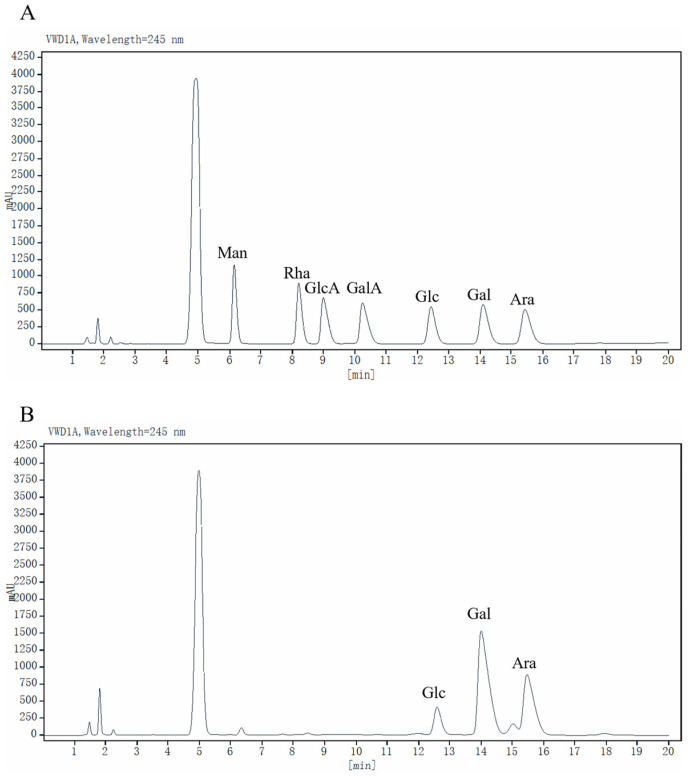
HPLC analysis of standard monosaccharides (**A**) and PEP-1-1 (**B**).

PMP pre-column derivation and HPLC analysis were used to detect the monosaccharide composition of PEP-1-1. The results are shown in Figure 1A,B. PEP-1-1 mainly contained Gal, Ara, Glc, and Man with a molar ratio of 52.61:32.89:12.08:1.50. The findings diverged from the monosaccharide compositions of the polysaccharide extracted from *Phyllanthus emblica*, as documented in prior studies. This variance could be attributed to differences in the extraction methods, purification procedures, or the employed methods and instruments for identification [28]. Galactose was the predominant monosaccharide group in PEP-1-1, making up the highest proportion among the identified monosaccharides. The proportion of glucuronic acid and galacturonic acid in PEP-1-1 was extremely low, which indicated that PEP-1-1 is a neutral polysaccharide.

### 2.3. FT-IR Analysis

The infrared spectrum exhibited characteristic absorption peaks corresponding to the O-H stretching vibration at 3402.43 cm^−1^ and C–H stretching vibrations at 2891.29 cm^−1^, which are typical signals associated with polysaccharides [29]. The absorption peak of 1375.24 cm^−1^ belonged to the variable angle vibration of C–H. Additionally, the presence of a pyranose ring with C–O–C and C–OH stretching vibration was confirmed by a strong absorption peak at 1047.34 cm^−1^ [30]. Small characteristic peaks at 900.75 and 813.96 cm^−1^ were observed in PEP-1-1, which showed that PEP-1-1 had both α-and β-type glycosidic linkages [31], respectively. Furthermore, the peak at 1604.77 cm^−1^ was attributed to the bound water [32]. The results are shown in Figure S2.

### 2.4. Surface-Enhanced Raman Spectroscopy Analysis

**Figure 2:**
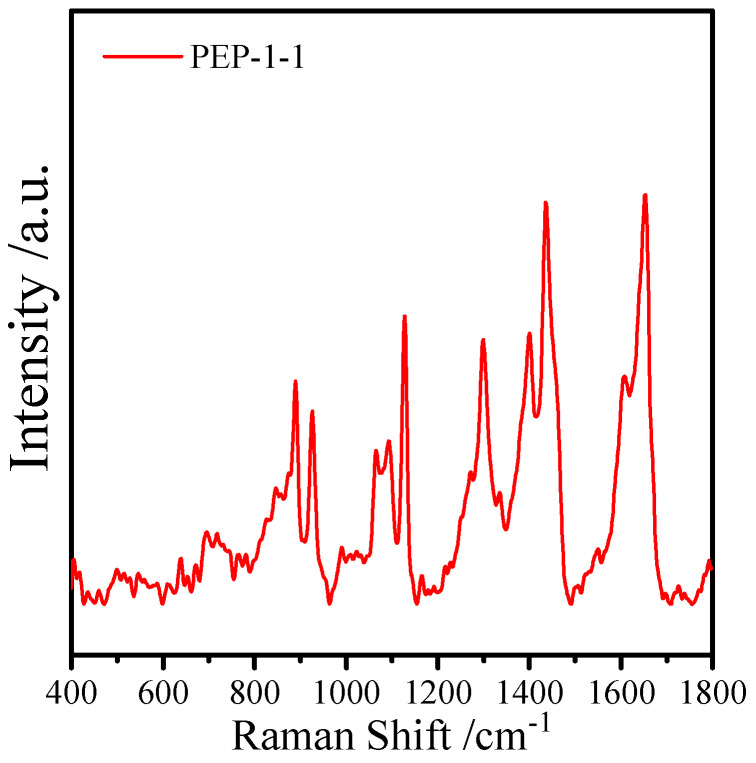
Surface-enhanced Raman spectroscopy spectrum of PEP-1-1.

The surface-enhanced Raman spectroscopy of PEP-1-1 is shown in Figure 2; significant polysaccharide signal peaks were observed within it, including C-C-O-C-O stretching vibration at 853 cm^−1^ [33], C-H stretching vibration at 925 cm^−1^ [34], C-O shear vibration at 1120 cm^−1^ [35], C-O-H bending vibration at 1300 cm^−1^ [36], C-H bending with some O-H contribution at 1410 cm^−1^, and CH_2_ stretching vibration at 1455 cm^−1^ were [37].

### 2.5. Methylation Analysis

Methylation serves as a potent and efficient approach for predicting the sugar linkage patterns of polysaccharides. Based on the retention time and mass spectrum information available in the CCRC Spectral Database (https://www.ccrc.uga.edu, accessed on 20 August 2023) and GC-MS analysis, PEP-1-1 was found to possess a minimum of four glycosidic linkages, including T-Araf, 1,5-Araf, 1,3,5-Araf, and 1,3-Galp (Table S2). Previous studies have shown that arabinogalactan had unique biological activities, such as immune activity, anticancer activity [38], and anti-aging activity [39]. The results suggest that antioxidant and anti-glycolipid metabolism disorder activities might also be due to large amounts of galactose and arabinose. A further deduction in the intricate structures of PEP-1-1 necessitated a detailed examination through an NMR analysis.

### 2.6. NMR Spectra Analysis

**Figure 3:**
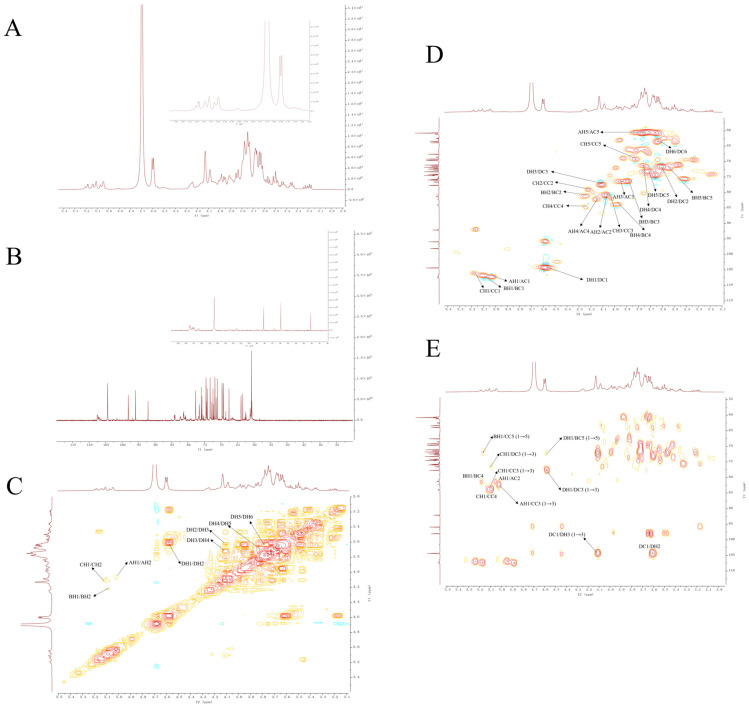
Nuclear magnetic resonance analysis of PEP-1-1. ^1^H-NMR spectrum (**A**), ^13^C-NMR spectrum (**B**), ^1^H-^1^H COSY spectrum (**C**), HSQC spectrum (**D**), and HMBC spectrum (**E**).

To delve deeper into the intricate structure of PEP-1-1, we conducted spectrum experiments on 1D NMR as well as 2D NMR. The majority of anomeric signals in PEP-1-1 were detected within the chemical shift range of δ 4.3–5.5 ppm for ^1^H-NMR and δ 90–120 ppm for ^13^C-NMR. Hydrogen signals indicative of the β-anomeric configuration were observed within the range of δ 4.5–4.8 ppm, while the corresponding carbon signals were identified in the δ 100–110 ppm range. In contrast, α-anomeric hydrogen signals were detected in the δ 4.8–5.8 ppm range, accompanied by corresponding carbon signals in the δ 90–100 ppm range [40]. Utilizing NMR assignments documented in the literature, the HSQC correlations identified at 5.03/107.31 and 5.17/106.35 were designated as H1/C1 for T-α-lAraf (labeled as residue A) and 1,5-α-l-Araf (B) [41], respectively. Another correlation of 5.10/106.90 was ascribed to H1/C1 for 1,3,5-α-l-Araf (C) [42], and the correlation of 4.60/104.50 was attributed to H1/C1 for 1,3-β-d-Galp (D) [40]. Concerning residue D, the COSY spectrum (Figure 3C) revealed cross signals at 4.60/3.62, 3.62/4.12, 4.12/3.75, 3.75/3.72, and 3.72/3.65 ppm, corresponding to H1/H2-H5/H6 assignments. Additionally, in the HSQC spectrum (Figure 3D), the correlation peaks at 4.60/104.50, 3.62/71.72, 4.12/77.50, 3.75/71.50, 3.72/73.21, and 3.65/60.68 ppm were designated as H1/C1-H6/C6 assignments, respectively [43,44]. Furthermore, the proton and carbon signals for other residues were assigned through the analysis of HSQC combined with ^1^H–^1^H COSY spectra. the H2/C2-H5/C5 values of residue A were 5.03/107.31, 4.07/81.03, 3.96/76.34, 4.15/82.07, and 3.75/60.99 ppm [45]. The H2/C2-H5/C5 values of residue B were 5.17/106.35, 4.24/81.34, 3.89/76.68, 3.98/83.83, and 3.98/83.83 [46]. The H2/C2-H6/C6 values of residue C were 5.10/106.90, 4.22/79.10, 4.03/82.06, 4.24/84.27, and 3.82/69.26 ppm [47]. Table 2 includes the NMR assignments for glycosidic residues A–D.

**Figure 4:**
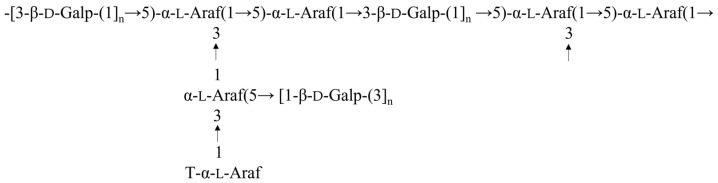
Primary chemical structure of PEP-1-1.

The analysis of the backbone structure and substitution sites was further conducted using the HMBC spectrum (Figure 3E). In summary, the correlation peak at 5.03/82.06 ppm signifies the connection between H1 of residue A and C3 of residue C, indicating the existence of a recurring unit [T-α-l-Araf-(1-3)-α-l-Araf(5)(1-)] within the polysaccharide structure. Furthermore, the correlation peak at 5.17/69.26 ppm suggests the connection of H1 of residue B to C5 of residue C, confirming the presence of a repeating unit [5-α-l-Araf-(1-5)-α-l-Araf(3)(1-)] in the polysaccharide structure. The correlation peak at 4.60/77.50 ppm and 4.12/104.50 ppm indicates the linkages of H1 of residue D to C3 of residue D and H3 of residue D to C1 of residue D. This suggests the presence of extensive repeating units [-3-β-d-Galp-(1-3)-β-d-Galp-(1-)] within the polysaccharide structure. The correlation peak at 5.10/82.06 ppm indicates the linkage of H1 of residue C to C3 of the same residue, suggesting the presence of a repeating unit [5-α-l-Araf-(3)(1-5)-α-l-Araf(3)(1-)] within the polysaccharide structure. In addition, Figure 3E illustrates additional cross peaks and the corresponding connected relationships among residues. There are still some inter-residual cross couplings of units in the picture. The cross peaks at 5.03/81.03, 5.17/83.83, 5.10/84.27, and 104.50/3.62 were ascribed to H1/C2 of A, H1/C4 of B, H1/C4 of C, and H2/C1 of D, respectively. Meanwhile, certain signals in the HMBC spectrum of PEP-1-1 were faint and remained partially unassigned, which was likely attributed to the low sugar residue content and the substantial Mw of PEP-1-1. Considering the established connections between residues, the majority of repeating units have been identified. Leveraging this determination, we can assemble these units in accordance with their connection relationships to deduce the potential structure of PEP-1-1 (Figure 4).

### 2.7. Congo Red Test Analysis

Congo red is a kind of acid dye that can interact with polysaccharides possessing a triple helical structure to form Congo red complexes. As shown in Figure S3, there was no observable shift towards longer wavelengths in the maximum absorption wavelength (λ max) of the Congo red–PEP-1-1 complex, indicating the absence of a triple helical structure in the polysaccharide. According to earlier studies, β-(1→3)-linked glycans with a molecular weight exceeding 90 kDa are more prone to adopting a triple helical conformation [48]. Overall, the associations between chain conformations and the bioactive properties of the polysaccharide remain unresolved and necessitate further comprehensive research.

### 2.8. SEM Analysis

Macromolecular microstructure and morphological characteristics can be observed using SEM [49]. In Figure S4, it can be seen that PEP-1-1 is an irregular network structure composed of short rod-shaped fragments, indicating that the regularity of the aggregates is not strong and that PEP-1-1 is an amorphous structure. Under high-power microscopy, it can be observed that the polysaccharide is an irregular granular aggregate, indicating that PEP-1-1 has no crystal structure and is an amorphous solid substance. This may be due to the low contents of glucuronic acid and protein macromolecules, fewer interaction points between particles, and a smaller interaction force, resulting in a low aggregation degree [50,51,52].

### 2.9. Antioxidant Activity of PEP-1-1 In Vitro

Oxidative stress is an imbalance between oxidation and antioxidant activities in the body, which can cause some important macromolecular substances in the organism to react with reactive oxygen species, lose their original physiological activity, and lead to cell damage and apoptosis [53].

**Figure 5:**
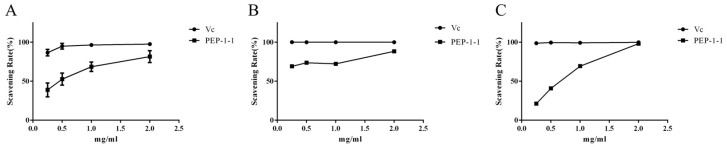
The antioxidant activities of PEP-1-1. Hydroxyl radical scavenging activity (**A**), superoxide anion radical scavenging activity (**B**), and ABTS scavenging activity (**C**).

Hydroxyl radicals are commonly recognized as potent oxidants. Upon entry into cells, they readily engage in reactions with various biological molecules [54]. As depicted in Figure 5A, the scavenging ability of PEP-1-1 against hydroxyl radicals was lower than that of VC at equivalent concentrations. When the concentration of PEP-1-1 increased from 0.25 mg/mL to 2 mg/mL, its clearance rate increased from 38.86 ± 8.87% to 81.64 ± 7.61%, indicating its dose dependence. At a safe cell concentration of 1 mg/mL, the hydroxyl radical scavenging rate of PEP-1-1 was 68.57 ± 6.09%, demonstrating good hydroxyl radical scavenging activity.

Superoxide anion radical is also a strong oxidant in the body, which will react with various organelles. In Figure 5B, when the concentration of PEP-1-1 increased from 0.25 mg/mL to 1 mg/mL, its free radical scavenging activity increased from 69.08 ± 1.18% to 72.37 ± 2.20%, indicating that PEP-1-1 has stable superoxide anion-free radical scavenging activity at low concentrations. However, when the concentration of PEP-1-1 increased from 1 mg/mL to 2 mg/mL, its scavenging activity increased to 88.42 ± 2.12%, showing a dose-dependent relationship.

To determine the total antioxidant capacity of a natural product, the ABTS method is commonly used [55]. In Figure 5C, when the concentration of PEP-1-1 increased from 0.25 mg/mL to 2 mg/mL, the scavenging activity of PEP-1-1 on ABTS free radicals increased from 21.21 ± 0.26% to 98.13 ± 0.26%, which is almost equal to that of the positive control drug Vc, indicating that the total reducing power of PEP-1-1 is dose-dependent. At a safe concentration of 1 mg/mL, the free radical scavenging activity of PEP-1-1 is 69.34 ± 0.40%, which is relatively ideal. Compared with the above experimental results, PEP-1-1 has good antioxidant activity and can achieve ideal comprehensive antioxidant capacity by clearing multiple free radicals.

### 2.10. Hypolipidemic Activity and Anti-Glycolipid Metabolism Disorder Activity of PEP-1-1

#### 2.10.1. Effects of PEP-1-1 on Cell Viability

HepG2 cells are commonly used as cell models to evaluate the lipid content. They are important cell models for studying fat synthesis and metabolism. Our study showed that, compared to the NC group, PEP-1-1 had no significant toxic effects. When PEP-1-1 was administered at a concentration of 1 mg/mL, the cell survival rate of HepG2 cells exceeded 90%, so subsequent experiments used this concentration as the dosing concentration. The results are shown in Figure S5.

#### 2.10.2. Effects of PEP-1-1 on Lipid Content in High-Fat HepG2 Cell Model Induced by OA

**Figure 6:**
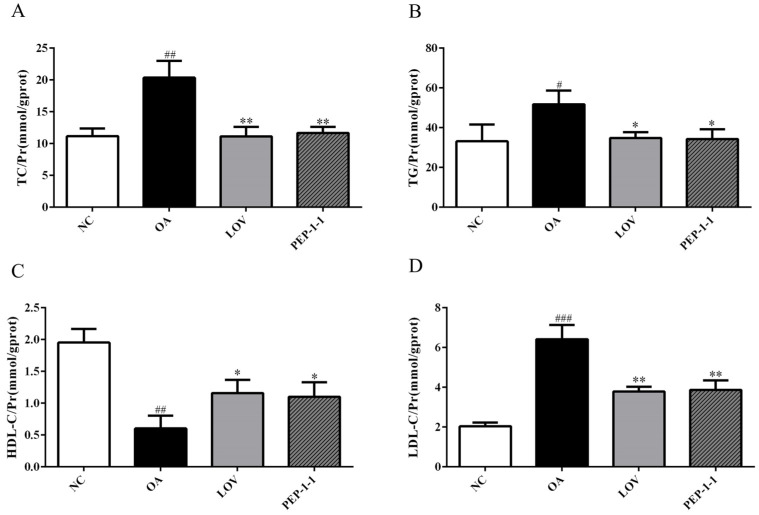
Effects of PEP-1-1 on contents of TC (**A**), TG (**B**), HDL-C (**C**), and LDL-C (**D**) in high-fat HepG2 cell model induced by OA; values shown as mean ± SD. (*n* = 3, ^#^ indicates *p* < 0.05 vs. NC group; ^##^ indicates *p* < 0.01 vs. NC group; ^###^ indicates *p* < 0.001 vs. NC group; * indicates *p* < 0.05 vs. model group; ** indicates *p* < 0.01 vs. model group).

The impact of PEP-1-1 on the lipid accumulation induced by OA in HepG2 cells is depicted in Figure 6. A high-fat cell model was successfully constructed by increasing the TC, TG, and LDL-C levels and decreasing the HDL-C levels in the OA group compared with those of the NC group. The positive control drug lovastatin could effectively improve lipid level abnormalities induced by OA; PEP-1-1 exhibited similar activity to lovastatin. Through OA induction, the contents of TG, TC, and LDL-C in HepG2 cells significantly increased from 33.14 ± 4.878 mmol/gprot, 11.16 ± 0.7119 mmol/gprot, and 2.038 ± 0.1101 mmol/gprot to 51.76 ± 3.967 mmol/gprot, 20.36 ± 1.514 mmol/gprot, and 6.414 ± 0.4151 mmol/gprot, respectively. The content of HDL-C decreased synchronously from 1.953 ± 0.1233 mmol/gprot to 0.6037 ± 0.1160 mmol/gprot, indicating the successful establishment of a high-fat model. After intervention with Lov and PEP-1-1, the abnormal lipid profile was improved, and the TG, TC, and LDL-C contents significantly decreased, reaching 34.86 ± 1.632 mmol/gprot and 34.25 ± 2.842 mmol/gprot, 11.13 ± 0.8662 mmol/gprot and 11.65 ± 0.5526 mmol/gprot, and 3.785 ± 0.1391 mmol/gprot and 3.868 ± 0.2786 mmol/gprot, respectively. HDL-C increased to 1.160 ± 0.1200 mmol/gprot and 1.102 ± 0.1309 mmol/gprot. In summary, PEP-1-1 has excellent hypolipidemic activity and can effectively improve lipid abnormalities induced by OA in HepG2 cells.

#### 2.10.3. Effects of PEP-1-1 on Lipid Content in High-Fat HepG2 Cell Model Induced by AGEs

**Figure 7:**
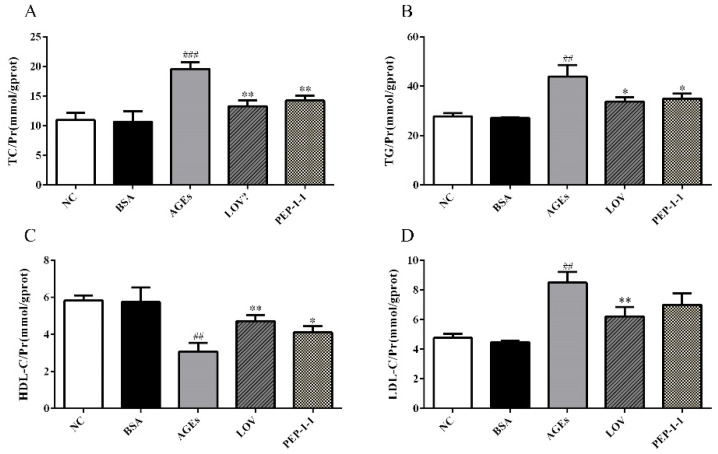
Effects of PEP-1-1 on contents of TC (**A**), TG (**B**), HDL-C (**C**), and LDL-C (**D**) in high-fat HepG2 cell model induced by AGEs; values are shown as mean ± SD. (*n* = 3, ^##^ indicates *p* < 0.01 vs. NC group; ^###^ indicates *p* < 0.001 vs. NC group; * indicates *p* < 0.05 vs. model group; ** indicates *p* < 0.01 vs. model group).

The generation of AGEs affects the normal function of proteins, promotes lipid peroxidation, and induces insulin resistance [56,57]. This experiment utilizes AGEs to induce the formation of a high-fat model in HepG2 cells, which associates glucose metabolism and lipid metabolism at the cellular level, enabling the expression of glucose and lipid metabolism disorders in the same cell model and forming a convenient and effective model for constructing glucose and lipid metabolism disorders. The effect of PEP-1-1 on AGE-induced lipid accumulation in HepG2 cells is shown in Figure 7. The lipid levels in AGE group cells were significantly abnormal, with increased the levels of TG, TC, and LDL-C, while the HDL-C level decreased. Under the intervention of Lov and PEP-1-1, the status of lipid abnormalities significantly improved. Through AGE induction, the contents of TG, TC, and LDL-C in HepG2 cells significantly increased from 27.84 ± 0.7262 mmol/gprot, 10.96 ± 0.6977 mmol/gprot, and 4.765 ± 0.1651 mmol/gprot to 43.90 ± 2.730 mmol/gprot, 19.59 ± 0.6546 mmol/gprot, and 8.492 ± 0.4186 mmol/gprot, respectively. The content of HDL-C decreased synchronously from 5.826 ± 0.1640 mmol/gprot to 3.077 ± 0.2749 mmol/gprot, indicating the successful establishment of a high-fat model. After intervention with Lov and PEP-1-1, the abnormal lipid profile was improved, and the TG, TC, and LDL-C contents significantly decreased, reaching 33.79 ± 1.006 mmol/gprot and 34.96 ± 1.151 mmol/gprot, 13.30 ± 0.5597 mmol/gprot and 14.26 ± 0.4846 mmol/gprot, and 6.216 ± 0.3649 mmol/gprot and 7.000 ± 0.4559 mmol/gprot, respectively. HDL-C increased to 4.725 ± 0.1903 mmol/gprot and 4.113 ± 0.1856 mmol/gprot. In summary, AGEs successfully correlated glucose metabolism with lipid metabolism and constructed a new cell model of glucose and lipid metabolism disorders. PEP-1-1 showed certain hypolipidemic activity in this model, which has potential development and utilization value.

## 3. Materials and Methods

### 3.1. Materials

*Phyllanthus emblica* fruits were sourced from Anqing, Anhui, China. Cellulose DEAE-52 was purchased from Solarbio Biochemical Co., Ltd. (Beijing, China). Amberlite FPA90 and Amberlite FPC3500 were purchased from Macklin (Shanghai, China). Penicillin-Streptomycin Solution was purchased from Beyotime (Shanghai, China). Detection kits for TC, TG, HDL-C, and LDL-C were purchased from Nanjing Jiancheng Biology Engineering Institute (Nanjing, China). All other chemical reagents were of analytical grade or chromatographic grade.

### 3.2. Extraction, Isolation, and Purification of Polysaccharides from Phyllanthus emblica

The preparation and purification of *Phyllanthus emblica* polysaccharides (PEPs) were carried out by ultrasound extraction and alcohol precipitation methods with some modifications [58]. First, we took about 100 g of dry powder of *Phyllanthus emblica* fruits and added it to distilled water at the solid–liquid ratio of 1:30 (*w*/*v*). Then, we placed the flask in an ultrasonic bath with a power setting of 100 W and a temperature of 60 °C for 6 h to obtain crude extract of *Phyllanthus emblica*. After filtration and concentration, Sevag reagent was added for deproteination. We added three times the volume of 95% ethanol to the aqueous extract for precipitation and left it overnight at 4 °C, allowing polysaccharides from *Phyllanthus emblica* to precipitate at the bottom of the solution. Finally, the redissolution of the precipitates was followed by dialysis using distilled water to yield a crude polysaccharide (cPEP) fraction after lyophilization.

About 10 g of cPEP was dissolved and centrifuged before loading onto an Amberlite FPA90Cl column connected in series with an Amberlite FPC3500H column. The columns were subjected to elution using a gradient of NaCl solutions (0, 0.5, and 1 M) at a flow rate of 10 mL/min, leading to the collection of three fractions (PEP-1, PEP-2, and PEP-3). The primary fraction (PEP-1) underwent further purification by being loaded onto a DEAE-52 column with gradient elution using a NaCl solution (0–0.5 M) at a flow rate of 0.6 mL/min. Each eluate was collected and analyzed using the phenol–sulfuric acid method at 490 nm. The main purified fraction of PEP-1 was collected and lyophilized after the dialysis of the eluate (Mw = 3500 kDa) and named PEP-1-1.

### 3.3. Homogeneity and Molecular Weight (Mw)

By using a gel chromatograph (GPC), the homogeneity and Mw of PEP-1-1 were assessed [59]. The TSKgel G5000PW_XL_ column (7.8 × 300 mm, 13 μm, Tosoh, Tokyo, Japan) was used on a Shimadzu LC-6A series apparatus equipped with a differential refractive index detector (Shimadzu, Kyoto, Japan) to measure the homogeneity and Mw of PEP-1-1. The mobile phase was ultrapure water flowing at 25 °C at 1 mL/min. An aqueous solution of the sample (PEP-1-1) was prepared and passed through a 0.22 μm membrane filter before being applied to the HPSEC system. Standard curves were prepared using dextran standards with different Mws.

### 3.4. Chemical Composition Analysis

The content of total sugar, protein, and uronic acid was measured using the phenol–sulfuric acid colorimetric method [60], Bradford method [61], and carbazole colorimetric method [62]. d-glucose, bovine serum albumin, and d-glucuronic acid were used as the standards, respectively.

### 3.5. Structural Characteristics of PEP-1-1

#### 3.5.1. Determination of Particle Size and ζ-Potential

A Malvern NanoZS90 laser particle size analytical instrument (Malvern, Malvern, UK) was used to investigate the particle size and ζ-potential of PEP-1-1. The particle size and ζ-potential measurements for PEP-1-1 were conducted three times in this segment to ensure accuracy and reliability.

#### 3.5.2. Monosaccharide Composition Assay

The monosaccharide composition analysis of PEP-1-1 involved the use of PMP pre-column derivatization and HPLC analysis [63]. Concisely, 10 mg of PEP-1-1 was hydrolyzed with trifluoroacetic acid (TFA) at 120 °C for 4 h. After removing the residual TFA with methanol under a reduced pressure, 200 μL of PMP and 200 μL of NaOH were added to derivatize at 70 °C for 1 h. Then, HCl was used to termination. The derivatized product was analyzed by HPLC equipped with UV detection(Agilent, Santa Clara, CA, USA) at 245 nm, eluted with KH_2_PO_4_-K_2_HPO_4_ (0.1 M, pH = 6.86) containing 18.5% acetonitrile at a flow rate of 1.0 mL/min. The monosaccharide standards were processed and analyzed as above.

#### 3.5.3. UV and FT-IR Analysis

The ultraviolet absorption spectrum of PEP-1-1 at the wavelength ranging from 200 nm to 400 nm was investigated using a UV-2550 spectrophotometer (Shimadzu, Kyoto, Japan). Then, a dried PEP-1-1 tablet mixed with an appropriate amount of KBr was analyzed using an FT-IR 650 spectrometer (Shimadzu, Kyoto, Japan) with a scanning range of 4000 to 400 cm^−1^.

#### 3.5.4. Surface Enhanced Raman Spectroscopy (SERS) Analysis

WITecalpha300R confocal Raman microscope (Witec, Ulm, Germany) was used to analyze PEP-1-1. In short, 1 mg/mL of PEP-1-1 solutions was prepared, and 2 μL of it was dropped onto silver plates with a power of 20 mw and a scanning time of 20 s for analysis.

#### 3.5.5. Methylation and GC-MS Analysis

The methylation analysis of PEP-1-1 was conducted according to the previously described method [64]. Briefly, 10 mg of PEP-1-1 was dissolved in 6 mL of DMSO and then reacted with 120 mg of dried NaOH powder and 0.9 mL of CHI_3_ under an argon gas atmosphere. Following the reaction, water was employed to halt the methylation process, and the methylated products were subsequently extracted three times using chloroform. The dried methylated reaction products were sequentially subjected to hydrolysis using 2 mol/L of trifluoroacetic acid (TFA). The resulting hydrolysate underwent reduction with NaBH_4_, followed by neutralization with acetic acid and acetylation with acetic anhydride. Ultimately, the analysis of partially methylated alditol acetates (PMAAs) was conducted using a 7890A-5975C GC-MS (Agilent, Santa Clara, CA, USA).

#### 3.5.6. NMR Analysis

PEP-1-1 powder was dissolved in deuterated water (D_2_O), followed by obtaining ^1^H-NMR, ^13^C-NMR, and 2D NMR spectra, including, HSQC, HMBC, and ^1^H-^1^H COSY, using a 600 MHz AV NEO NMR spectrometer equipped with a cryogenic probe (Brucker, Karlsruhe, Germany).

#### 3.5.7. Congo Red Test

The Congo red test was used to investigate whether PEP-1-1 possessed a triple helical structure [65]. After mixing 1 mL of a 1 mg/mL PEP-1-1 solution with 1 mL of a 200 mmol/L Congo red solution, the resulting mixture was added to varying gradients of NaOH solution. The solution mixture was examined across the 200–800 nm spectrum to pinpoint the wavelength at which maximum absorption occurred.

#### 3.5.8. Scanning Electron Microscopy (SEM) Analysis

The polysaccharide powder was coated with conductive gold before being examined using a scanning electron microscope system at magnifications of 500× and 1000× with a working voltage of 5.0 kV.

### 3.6. Antioxidant Effects of PEP-1-1 In Vitro

#### 3.6.1. Determination of Scavenging Capacity of Hydroxyl Radicals

The hydroxyl radical scavenging capacity of PEP-1-1 was determined using Lin’s method [66]. The test tube was supplied with 1 mL of PEP-1-1 at different concentrations. Then, 1 mL of FeSO_4_, 1 mL of salicylic acid–ethanol solution, and 1 mL of H_2_O_2_ solution were added and mixed. Following an incubation period of 30 min at 37 °C, the absorbance of the mixture was measured at 510 nm. Vc served as the positive control.

#### 3.6.2. Determination of Scavenging Capacity of Superoxide Anion Radicals

The superoxide anion radical scavenging capacity of PEP-1-1 was determined using Chen’s method [30]. At various concentrations, 1 mL of PEP-1-1 was introduced into a test tube along with a mixture of 3.9 mL of ultrapure water and 4.5 mL of 50 mmol/L Tris-HCl buffer (pH = 8.2). The combination was allowed to incubate at 25 °C for 20 min. Subsequently, 0.5 mL of 50 mmol/L pyrogallic acid was introduced to the solution, and after thorough shaking, absorbance readings were recorded at 325 nm for each group.

#### 3.6.3. Determination of Scavenging Capacity of ABTS Radicals

The ABTS radical scavenging capacity of PEP-1-1 was determined using Li’s method [67]. The prepared ABTS^+^ solution was diluted to the absorbance of 0.70 ± 0.02 at 734 nm. An amount of 1 mL of PEP-1-1 was mixed well in the test tube at different concentrations with 6 mL of ABTS^+^ solution. The absorbance of the mixture was recorded at 734 nm following a 10 min reaction at 25 °C.

All three radical scavenging rates were determined using the following equation:

Scavenging activity (%) = [1 − (A_1_ − A_2_)/A_0_] × 100%

where

A_0_: Absorption of samples replaced by ultrapure water;
A_1_: Absorption of sample groups;
A_2_: Absorption of sample groups’ background.

### 3.7. Hypolipidemic Activity and Anti-Glycolipid Metabolism Disorder Activity of PEP-1-1

#### 3.7.1. Preparation of AGEs

The experiments were conducted following the methodology outlined in our earlier study [68]. Briefly, BSA, glucose, and sodium azide were combined in a phosphate buffer, and the resulting mixture underwent a 3-month incubation period at 37 °C. Glycosylation product fluorescence was measured at a 370 nm excitation wavelength and a 440 nm emission wavelength after being diluted to 10 mL with ultrapure water.

#### 3.7.2. Cell Culture

HepG2 cells were provided by the Department of Pharmacology in Harbin Medical University and grown in DMEM high-glucose medium containing 10% FBS (fetal bovine serum). Cell cultures were kept in an incubator at 37 °C with a gas mixture of 5% carbon dioxide and 95% air. Passage of cells at 80–90% confluence was performed using 0.5% trypsin.

#### 3.7.3. Cell Viability Assay

HepG2 cells were plated on a 96-well plate at a density of 1 × 10^3^ cells per well and allowed to adhere for 24 h before being exposed to PEP-1-1 treatment. After 48 h, each well received an addition of 10 μL of CCK-8, followed by incubation at 37 °C for 4 h, and the absorbance was measured at 450 nm. Cell viability was quantified as the percentage change in absorbance values compared to the negative control (NC) group within the treatment group.

#### 3.7.4. Effect of PEP-1-1 on Lipid Content in High-Fat HepG2 Cell Model Induced by OA

We took HepG2 cells in good condition, added trypsin digestion solution to digest the cells, counted and adjusted the cell density to 1 × 10^6/^mL, inoculated 12 mL in a 24-well plate, and divided them into an NC group, model group, lovastatin-positive control group, and PEP-1-1 treatment group; each group was set to 3 wells. After culturing for 12 h in the dark, we removed the old culture medium, and then the NC control group was added with 500 µL of medium, the model group and the positive control group were treated with 500 µL of 0.5 mM oleic acid modeling solution and 20 µM lovastatin, and 1.0 mg/mL of PEP-1-1 was added to the treatment group. They continued to incubate for 48 h in the incubator. We determined the contents of TC, TG, HDL-C, LDL-C, and protein in each well sample according to the method shown in the kit instructions.

#### 3.7.5. Effect of PEP-1-1 on Lipid Content in High-Fat HepG2 Cell Model Induced by AGEs

As described above, HepG2 cells were digested and seeded in a 24-well plate and then incubated for 12 h until the cell density reached 80–90%, and then we divided them into an NC group, BSA group, AGE group, lovastatin-positive control group, and PEP-1-1 treatment group; each group was set to 3 wells. An amount of 1.0 mg/mL of AGEs was used to induce HepG2 cells to form a high-fat model. The treatment group added 500 µL of 1.0 mg/mL of PEP-1-1 to process the cells. The experiment utilized 20 μM of lovastatin as the positive control, the medium was used instead of AGEs in the NC group, and the BSA group was set up to eliminate potential interference from BSA. After 48 h of incubation, the contents of TC, TG, LDL, HDL, and protein were measured using the method shown in the kits.

### 3.8. Statistical Analysis

Experimental data were presented as mean ± standard deviation (mean ± SD), and statistical analysis was performed using GraphPad Prism 7 software. The experimental results between the groups were compared using the *t* test method, and the difference was recorded; *p* < 0.05 was deemed statistically significant.

## 4. Conclusions

In this study, a purified polysaccharide of *Phyllanthus emblica* named PEP-1-1 was obtained. In addition to analyzing its chemical structure, its anti-glycolipid metabolism disorder ability was studied for the first time. PEP-1-1 is a heteropolysaccharide with little uronic acid. The particle size and ζ-potential of PEP-1-1 are 1093 nm and −4.22 mV, respectively. The monosaccharide composed Gal, Ara, Glu, and Man with a molar ratio of 52.61:32.89:12.08:1.50. PEP-1-1 has a molecular mass of 29.26 kDa, and its backbone is mainly composed of -3)-β-d-Galp-(1-,-5)-α-l-Araf(3)(1- and -5)-α-l-Araf(1-residues. The lateral chains are attached to the primary chain via the O-1 and O-3 atoms of arabinose. A Congo red test confirmed that PEP-1-1 does not have a triple helical structure. The results show that PEP-1-1 had a strong effect on decreasing the TC, TG, and LDL-C levels and increasing the HDL-C level in OA-induced high-fat HepG2 cells. In addition, PEP-1-1 also showed a good effect in antioxidant activity in vitro. We designed a high-fat HepG2 cell model induced by AGEs to comprehensively evaluate the anti-glycolipid metabolism disorder activity of PEP-1-1. With the intervention of PEP-1-1, the TC, TG, and LDL-C levels decreased, and the HDL-C level increased in AGE-induced high-fat HepG2 cells, also indicating that PEP-1-1 has excellent potential to be developed as an anti-glycolipid metabolism disorder drug.

In summary, considering the financial costs and based on the aforementioned points, we believe that PEP-1-1 has the potential to serve as a drug candidate for treating glycolipid metabolic disorders and be used as a nutritional regulator and food additive. This provides a theoretical basis for the further development and utilization of *Phyllanthus emblica*.

## Figures and Tables

**Table 1:** The chemical composition and molecular properties of PEP-1-1.

Polysaccharide	Chemical Composition			Molecular Properties	
	Total Sugar	Protein	Uronic Acid	Particle Size	ζ-Potential
PEP-1-1	91.23%	0.35%	6.22%	1093 nm	−4.22 mV

**Table 2:** Chemical shifts of main residues of PEP-1-1.

Code	Residues	Chemical Shifts (ppm)					
		H1/C1	H2/C2	H3/C3	H4/C4	H5/C5	H6/C6
A	T-α-l-Araf-(1-	5.03/107.31	4.07/81.03	3.96/76.34	4.15/82.07	3.75/60.99	
B	-5-α-l-Araf-(1-	5.17/106.35	4.24/81.34	3.89/76.68	3.98/83.83	3.54/72.04	
C	-3,5)-α-l-Araf-(1-	5.10/106.90	4.22/79.10	4.03/82.06	4.24/84.27	3.82/69.26	
D	-3-β-d-Galp-(1-	4.60/104.50	3.62/71.72	4.12/77.50	3.75/71.50	3.72/73.21	3.65/60.68

## Data Availability

The raw data involved in the study can be queried from the corresponding author.

## Supplementary Materials

The following supporting information can be downloaded at: https://www.mdpi.com/article/10.3390/molecules29081751/s1, Figure S1: Flow chart of *Phyllanthus emblica* polysaccharide extraction, separation, and purification of PEP (A); elution curve of PEP-1 on DEAE-52 column (B); the HPSEC chromatogram of PEP-1-1 (C); the UV chromatogram of PEP-1-1 (D); Table S1: The yield and productivity of purified products; Figure S2: FT-IR spectrum of PEP-1-1; Figure S3: Congo red test experimental results of PEP-1-1; Figure S4: Scanning electron micrographs of PEP-1-1 at magnification of 500× (A), (B) and 1000× (C); Figure S5: Effects of PEP-1-1 on the cell viability; Table S2: Linkage patterns analysis of PEP-1-1.
