# Supplementary material for: Exploration of Polysaccharides from Phyllanthus emblica: Isolation, Identification, and Evaluation of Antioxidant and Anti-Glycolipid Metabolism Disorder Activities
Source: Molecules. 2024 Apr 12;29(8):1751. doi: 10.3390/molecules29081751 (PMC11052227; doi:10.3390/molecules29081751)
Supplement: Supplementary file 1 [file molecules-29-01751-s001.zip › molecules-2949820-supplementary.pdf]

Supplementary materials

Study on Isolation, Identification, Antioxidant and Anti-Glycolipid Metabolism Disorder Activities of Polysaccharide from *Phyllanthus emblica*

Peng Guo[1], Meng Chen[1], Wenzhao Wang, Qiuyun Li, Xinyu Chen, Jiayue Liang, Yiyang He, Yanli Wu*

Department of Organic Chemistry, College of Pharmacy, Harbin Medical University, Harbin, Heilongjiang 150081, China

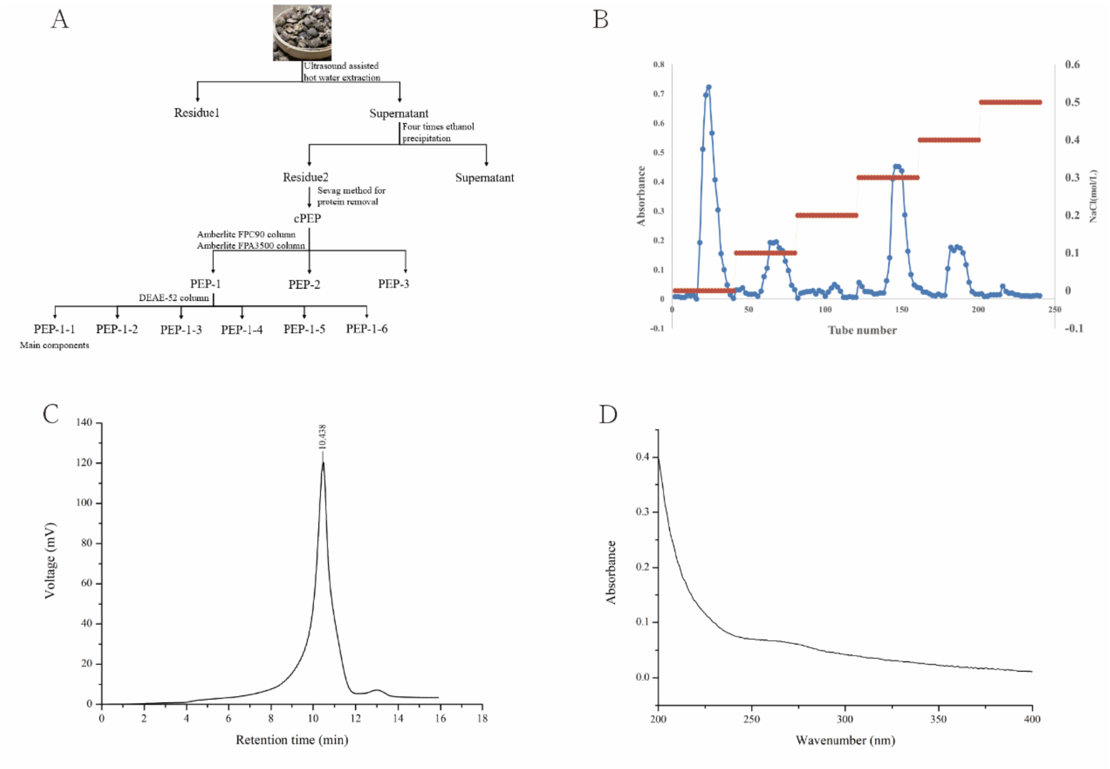

Fig.S1 Flow chart of *Phyllanthus emblica* polysaccharide extraction, separation, and purification of PEP (A); elution curve of PEP-1 on DEAE-52 column (B); the HPSEC chromatogram of PEP-1-1 (C); the UV chromatogram of PEP-1-1 (D).

Table S1.

The yield and productivity of purified products

Name	Eluent	The yield of the product (g)	The productivity of the product (%)
PEP-1	H_2O	1.2918	12.92
PEP-2	0.5 M NaCl	0.6244	6.24
PEP-3	1 M NaCl	0.2514	2.51
PEP-1-1	H_2O	0.136	11.33
PEP-1-2	0.1 M NaCl	0.054	4.50
PEP-1-3	0.2 M NaCl	0.018	1.50
PEP-1-4	0.3 M NaCl	0.026	2.17
PEP-1-5	0.4 M NaCl	0.014	1.21
PEP-1-6	0.5 M NaCl	0.006	0.53

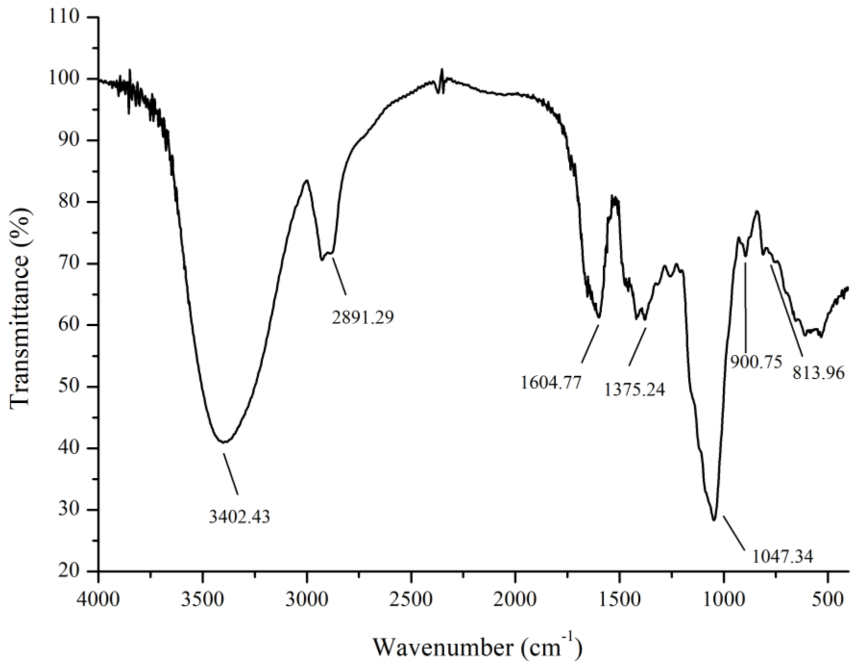

Fig.S2 FT-IR spectrum of PEP-1-1.

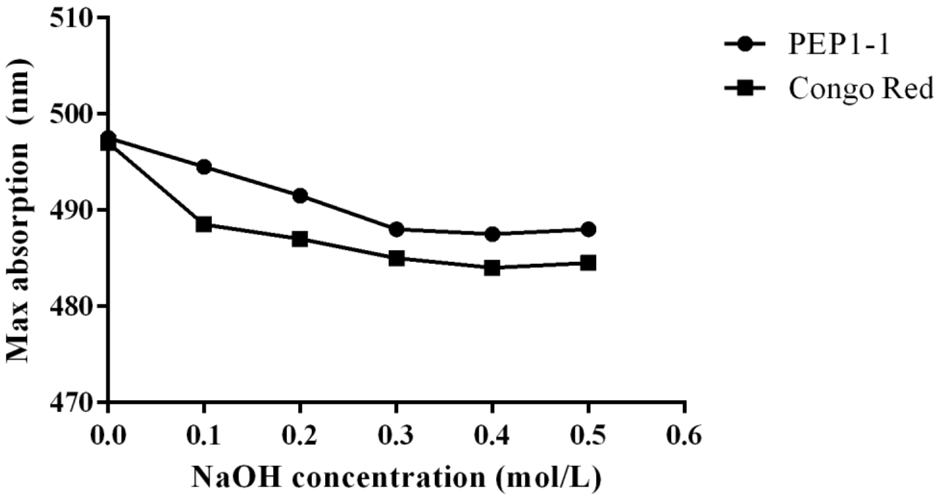

Fig.S3 Congo red test experimental results of PEP-1-1.

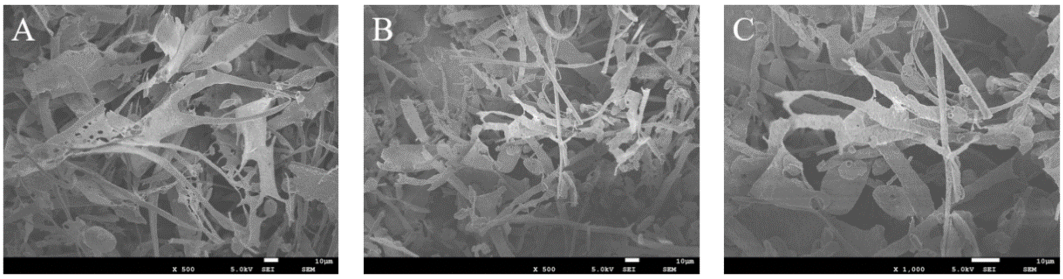

Fig.S4 Scanning electron micrographs of PEP-1-1 at magnification of 500 × (A), (B) and 1000 × (C).

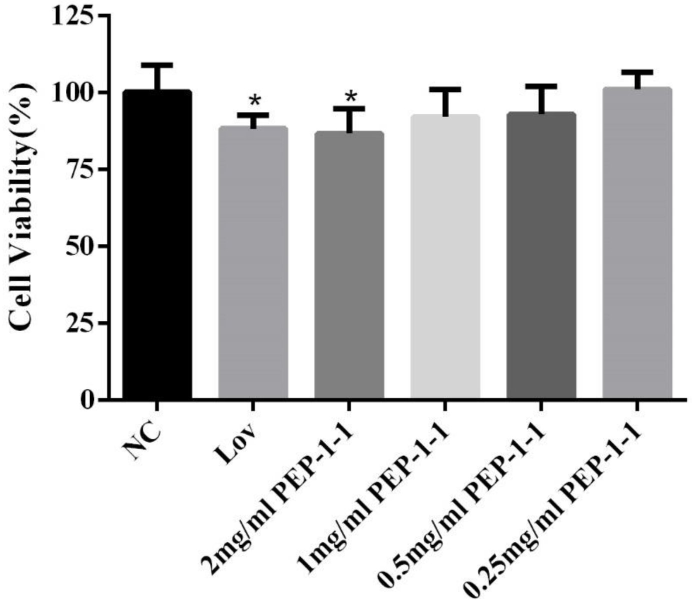

Fig.S5 Effects of PEP-1-1 on the cell viability. (n=6, * indicated $p<0.05$ vs. NC group.)

Table S2.

Linkage patterns analysis of PEP-1-1

Peak	RT (min)	Partially methylated alditol acetate (PMAA)	Mass fragments (*m/z*)	Linkage types
1	21.358	1,4-Di-*O*-acetyl-2,3,5-tri-*O*-methyl-D-arabinitol	58,71,87,101,117,129,161	T-α-L-Araf-(1-
2	24.893	1,4,5-Tri-O-acetyl-1-deuterio-2,3-di-O-methyl-D-arabinitol	58,71,87,101,117,129,161,189	-5-α-L-Araf-(1-
3	26.621	1,2,5-Tri-O-acetyl-1-deuterio-3,4,6-tri-O-methyl-D-galactitol	58,71,87,101,117,129,142,161,191,203	-3,5)- α-L Araf -(1-
4	28.192	1,3,5-Tri-O-acetyl-1-deuterio-2,4,6-tri-O-methyl-D-galactitol	58,71,87,101,117,129,161,173,203,233	-3-β-D-Galp-(1-
